# Supplementary figures and images for: The Loading of Epigallocatechin Gallate on Bovine Serum Albumin and Pullulan-Based Nanoparticles as Effective Antioxidant
Source: Foods. 2022 Dec 16;11(24):4074. doi: 10.3390/foods11244074 (PMC9777656; doi:10.3390/foods11244074)

### Supporting Information

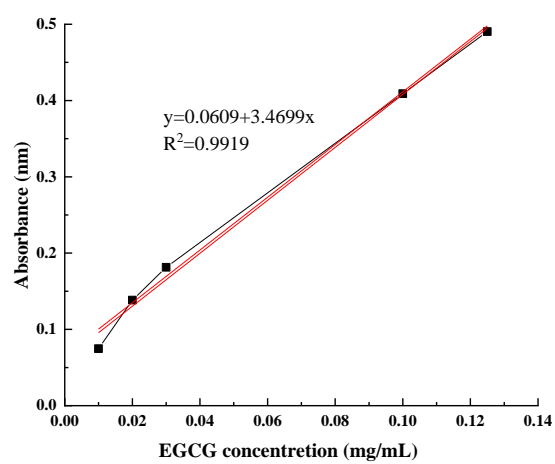

**Figure S1** Standard curve of EGCG

Supplement: Supplementary file 1 [file foods-11-04074-s001.zip › foods-1995607-supplementary.pdf]
